# Altering α-dystroglycan receptor affinity of LCMV pseudotyped lentivirus yields unique cell and tissue tropism

**DOI:** 10.1186/1479-0556-9-8

**Published:** 2011-04-08

**Authors:** Douglas E Dylla, Litao Xie, Daniel E Michele, Stefan Kunz, Paul B McCray

**Affiliations:** 1 Genetics Ph.D. Program, Program in Gene Therapy, 240 EMRB, The University of Iowa Roy J. and Lucille A. Carver College of Medicine, The University of Iowa, Iowa City, IA 52242 USA; 2 Department of Pediatrics, 240 EMRB, The University of Iowa Roy J. and Lucille A. Carver College of Medicine, The University of Iowa, Iowa City, IA 52242 USA; 3 Microbiology, 240 EMRB, The University of Iowa Roy J. and Lucille A. Carver College of Medicine, The University of Iowa, Iowa City, IA 52242 USA; 4 Molecular and Integrative Physiology, University of Michigan, 7771 Med Sci II, Ann Arbor, MI 48109, USA; 5 Institute of Microbiology, University Hospital Center and University of Lausanne, CH-1011, Lausanne, Switzerland

## Abstract

**Background:**

The envelope glycoprotein of lymphocytic choriomeningitis virus (LCMV) can efficiently pseudotype lentiviral vectors. Some strains of LCMV exploit high affinity interactions with α-dystroglycan (α-DG) to bind to cell surfaces and subsequently fuse in low pH endosomes. LCMV strains with low α-DG affinity utilize an unknown receptor and display unique tissue tropisms. We pseudotyped non-primate feline immunodeficiency virus (FIV) vectors using LCMV derived glycoproteins with high or low affinity to α-DG and evaluated their properties *in vitro *and *in vivo*.

**Methods:**

We pseudotyped FIV with the LCMV WE54 strain envelope glycoprotein and also engineered a point mutation in the WE54 envelope glycoprotein (L260F) to diminish α-DG affinity and direct binding to alternate receptors. We hypothesized that this change would alter *in vivo *tissue tropism and enhance gene transfer to neonatal animals.

**Results:**

In mice, hepatic α- and β-DG expression was greatest at the late gestational and neonatal time points. When displayed on the surface of the FIV lentivirus the WE54 L260F mutant glycoprotein bound weakly to immobilized α-DG. Additionally, LCMV WE54 pseudotyped FIV vector transduction was neutralized by pre-incubation with soluble α-DG, while the mutant glycoprotein pseudotyped vector was not. *In vivo *gene transfer in adult mice with either envelope yielded low transduction efficiencies in hepatocytes following intravenous delivery. In marked contrast, neonatal gene transfer with the LCMV envelopes, and notably with the FIV-L260F vector, conferred abundant liver and lower level cardiomyocyte transduction as detected by luciferase assays, bioluminescent imaging, and β-galactosidase staining.

**Conclusions:**

These results suggest that a developmentally regulated receptor for LCMV is expressed abundantly in neonatal mice. LCMV pseudotyped vectors may have applications for neonatal gene transfer.

**Abbreviations:**

Armstrong 53b (Arm53b); baculovirus *Autographa californica *GP64 (GP64); charge-coupled device (CCD); dystroglycan (DG); feline immunodeficiency virus (FIV); glycoprotein precursor (GP-C); firefly luciferase (Luc); lymphocytic choriomeningitis virus (LCMV); nuclear targeted β-galactosidase (ntLacZ); optical density (OD); PBS/0.1% (w/v) Tween-20 (PBST); relative light units (RLU); Rous sarcoma virus (RSV); transducing units per milliliter (TU/ml); vesicular stomatitis virus (VSV-G); wheat germ agglutinin (WGA); 50% reduction in binding (C_50_).

## Background

Arenaviruses are a family of single-stranded, enveloped, bisegmented RNA viruses that include the Old World arenaviruses lymphocytic choriomeningitis virus (LCMV) and Lassa fever virus, and the New World arenaviruses Machupo, Junin, and Guanarito. LCMV glycoproteins can pseudotype retroviral and HIV-based lentiviral vectors facilitating studies of virus biology and gene transfer [1-3]. Encoded by the small RNA fragment, the arenavirus glycoprotein precursor (GP-C) is post-translationally cleaved to yield GP1 and GP2. GP1 is believed to be responsible for receptor binding followed by a pH-dependent fusion step mediated by GP2 [4,5]. Several Old World arenaviruses utilize α-dystroglycan (α-DG) as a viral receptor [6]. LCMV is the prototypic Old World arenavirus, with different strains displaying either high or low affinity for α-DG. Alpha-DG expression is developmentally regulated, displaying the highest expression levels in developing tissues involved in basement membrane assembly [7]. Expression rises during embryonic stages, peaks in the newborn, and diminishes in adult tissues [8,9]. Here we develop and investigate the use of LCMV envelope glycoproteins with high or low α-DG affinity for lentiviral gene transfer applications.

α-DG is a ubiquitously expressed, versatile, evolutionarily conserved cell surface receptor that links the extracellular matrix with the cytoskeleton, making it an ideal target for pathogen binding [7,10]. The dystroglycan complex is transcribed as a precursor peptide that undergoes post-translational cleavage to produce α- and β-DG. Noncovalently linked, α- and β-DG act as peripheral and transmembrane proteins, respectively. Interestingly, α-DG usage correlates with persistent infection, disease kinetics, and tropism [11]. The immunosuppressive LCMV isolates WE54, LCMV Cl13, and Traub efficiently target antigen presenting cells (dendritic cells) in the spleen and perturb their ability to present antigen to T cells and B cells, resulting in a generalized immunosuppression of the host allowing viral persistence. These three LCMV strains bind to α-DG with high affinity, unlike non-immunosuppressive LCMV variants Armstrong 53b, CD4-1, CD8-4 and WE2.2, which demonstrate low affinity. LCMV Arm53b and WE2.2 replicate primarily in the splenic red pulp and infection is rapidly resolved. The tropism differences between LCMV strains, as well as significant infection of DG^-/- ^ES cells by non-immunosuppressive LCMV variants [12] suggest that an alternate and currently unidentified viral receptor is utilized by LCMV strains with low affinity for α-DG [13].

LCMV Arm53b and clone13 are nearly genetically identical with the exception of two amino acid changes, one occurring in the viral polymerase encoded by the large RNA fragment and the other at position 260 of GP1 [14]. LCMV WE54 and WE2.2 are also genetically similar with only one amino acid difference at position 153 of GP1. Genetic and phenotypic comparison of the New World arenavirus and LCMV variants led to the observation that amino acid 260 of GP1 plays an important role in their ability to utilize α-DG [12,15]. Spiropoulou et al. generalized that a leucine or isoleucine residue at position 260 was required for high α-DG affinity, while bulky aromatic residues such as phenylalanine or tyrosine generally resulted in low affinity [16]. A similar change in affinity was observed between WE54 and WE2.2, the result of a serine to phenylalanine mutation at position 153 of GP1. Proper glycosylation, specifically O-mannosylation [17], and modifications of LARGE glycosyltransferase [18] also play important roles in receptor recognition by LCMV. However, LCMV isolates can transduce α-DG^-/- ^mouse ES cells with reduced efficacy, indicating that α-DG dependence is not absolute [3,11,19]. Utilization of an alternate receptor cannot be ruled out as a possibility.

Among the factors potentially limiting the utility of pseudotyped vectors are low vector titers, envelope instability following ultracentrifugation, glycoprotein cytotoxicity, and limited tropism. Able to withstand ultracentrifugation, LCMV glycoproteins yield MLV and HIV vector titers similar to the widely used amphotropic and VSV-G envelopes [1-3]. Stable cell lines constitutively expressing the WE54 envelope have been generated, demonstrating that the LCMV envelope exhibits little cytotoxicity in comparison to the VSV-G envelope [1]. Cannon and colleagues previously demonstrated successful pseudotyping of MLV-based retroviral vectors with the Armstrong 53b (Arm53b) envelope glycoprotein, and generated the F260L mutation in the Arm53b GP1 to generate a clone 13-like envelope with high α-DG affinity [3]. They successfully used these and other pseudotypes to investigate receptor use and α-DG affinity among several Old and New World arenavirus envelopes *in vitro *[3]. In this report we show that LCMV envelopes efficiently pseudotype a non-primate FIV lentiviral vector and maintain the entry properties seen in wild-type arenaviruses. Furthermore, we modified the WE54 LCMV envelope with the GP1 mutation L260F and altered vector α-DG affinity. Here we investigate the expression of α- and β-DG in the liver at pre- and postnatal time points and document the *in vivo *tissue tropisms of these LCMV pseudotypes in neonatal and adult mice. We hypothesized that a FIV vector pseudotyped with a LCMV envelope glycoprotein with low α-DG binding affinity would yield unique *in vivo *tissue tropism and enhance gene transfer efficiency in neonatal animals.

## Methods

### Glycoprotein enrichment and immunoblotting

Cell surface glycoproteins were isolated for quantification of dystroglycan expression as previously described [20]. Day 18 embryos or postnatal mice were collected from timed pregnant BALB/c mice, and the livers pooled from the entire litter. Pooled livers (~200 mg tissue per sample) were homogenized in Tris buffered saline, pH 7.5 +1.0% TX-100 containing a cocktail of protease inhibitors and the protein was quantified by DC assay (BioRad). The resulting homogenate was centrifuged at 4000 g for 15 minutes. A 50% suspension of wheat germ agglutinin (WGA) agarose beads were then added (100 μl of packed beads per 100 mg of tissue) and incubated at 4°C with end over end rotation. The beads were washed 3X with 10 volumes of Tris buffered saline, pH 7.5 + 0.1% TX-100. The beads were eluted by boiling for 5 minutes in 1X Laemlli SDS sample buffer. Protein samples were separated on 3-15% SDS-PAGE, transferred to PVDF membranes, and blotted with the monoclonal antibody IIH6 recognizing the glycosylated form of α-DG (Upstate), or a polyclonal antibody against β-DG (Santa Cruz). Western blots were developed with peroxidase conjugated secondary antibodies, ECL detection (Pierce) and imaged on a Fluorchem imaging station (Alpha Innotech).

### LCMV envelope mutagenesis

Quikchange site-directed mutagenesis (Stratagene, 200518) was used to create a point mutation in the LCMV WE54 glycoprotein plasmid (Genbank Accession AJ318512), a kind gift of W. R. Beyer described previously [1,21]. PAGE-purified primers (LCMV-L260F+, 5' GGAAAAGACAAAGTTTTTCACTAGGAGACTTGCAGGC 3', and LCMV-L260F-, 5' GCCTGCAAGTCTCCTAGTGAAAAACTTTGTCTTTTCC 3') were used in the PCR-based mutagenesis to generate a leucine to phenylalanine mutation at residue 260 of LCMV GP1, thus creating the envelope construct LCMV L260F. The L260F mutation was confirmed by sequencing.

### Vector production

The second-generation FIV vector used in this study was reported previously [22,23]. FIV vectors expressed the firefly luciferase (Luc) cDNA under the control of a Rous sarcoma virus (RSV) promoter or nuclear targeted β-galactosidase (ntLacZ) directed by the CMV promoter. Envelopes utilized in this study include the glycoproteins from Indiana strain vesicular stomatitis virus (VSV-G), baculovirus *Autographa californica *GP64 (GP64) [24], LCMV WE54 (also referred to as LCMV-GP(WE-HPI)) [21], and LCMV L260F. Pseudotyped FIV vector particles were generated by transient transfection of plasmid DNA as described previously [22]. Pseudotyped viruses expressing β-gal were visually titered on HT1080 cells (ATCC, CCL-121) following limiting dilutions of 250-fold centrifuge concentrated supernatants. Luciferase expressing vectors were titered by quantitative PCR following limiting dilution on HT1080 cells and by RT activity as described [25,26]. Lentiviral vectors for *in vivo *experiments were resuspended in 4% (w/v) α-lactose buffer [23].

### Inhibition of endosomal acidification

A549 cells (ATCC, CCL-185) were pretreated with the carboxylic ionophore monensin (8 μM) (Sigma, M5273) [27,28] or the weak base ammonium chloride (10 mM). Pretreatments were applied 1 hour prior to vector transduction at 4°C. The media were changed and FIV vectors pseudotyped with LCMV WE54, VSV-G, or MuLV amphotropic envelopes were applied at an MOI of 10. Viruses were incubated on the cells for 30 minutes at 37°C, then fresh media was replaced. Control cultures received vehicle treatment only. Four days later, gene transfer efficiency was assessed using Galacto-Light Plus beta-Galactosidase Reporter Gene Assay System (Applied Biosystems, BL300P) and normalized for total protein by Lowry assay.

### Binding of FIV pseudotypes to α-DG

α-DG was isolated from skeletal muscle [29], diluted to a concentration of 10 μg/ml in PBS and immobilized in 96-well EIA/RIA high-bond microtiter plates (Sigma-Aldrich, CLS3366). Following a 2-hour immobilization at 25°C, wells were washed 3 times with PBS. Non-specific binding was blocked by adding 200 μl/well 1% (w/v) BSA/PBS and incubating for 1 hour at 25°C. Wild-type LCMV isolates WE54 and WE2.2 were produced in BHK21 cells (ATCC, CCL-10), precipitated with PEG, purified on a renografin gradient by ultracentrifugation, and resuspended in 1% BSA/PBS yielding 10^7 ^pfu/ml. FIV pseudotyped with LCMV WE54 and L260F was prepared and concentrated as stated previously [23], then diluted to 10^7 ^transducing units per milliliter (TU/ml). Viruses were incubated on immobilized α-DG for 12 hours at 6°C on an orbital shaker (60 rpm) followed by 3 washes with PBS/0.1% (w/v) Tween-20 (PBST).

### Detection of bound virus using an ABC detection system

The primary antibody for detection of bound LCMV was mAb 83.6 anti-GP2 [5], purified IgG, 20 μg/ml in 1% BSA/PBS. Primary antibody was incubated for 2 hours at 6°C followed by 3 washes of PBST. Biotinylated goat anti-mouse IgG (1:500) secondary antibody in 1% (w/v) BSA/PBS was added for 1 hour at 25°C. Wells were washed 3 times with PBST. Steptavidin coupled to peroxidase (1:500) was added for 1 hour at 25°C followed by 3 washes with PBST. Detection using ABTS [2,2'-azinobis (3-ethylbenzthiazolinesulfonic acid)] substrate allowed for optical density at 405 nm (OD_405_) to be recorded in an ELISA reader.

### Blocking of transduction of LCMV and FIV pseudotypes with soluble α-DG

200 PFU of wild-type LCMV or 200 TU of FIV-LCMV β-gal pseudotypes were diluted in OPTIMEM, 2% (v/v) FBS with the indicated amounts of purified α-DG or BSA for 1 hour on ice. The inoculum was added to 90% confluent cultures of HEK293H cells (ATCC, CRL-1573) in 8-well LabTeks plates (Nunc) and incubated for 45 minutes at 37°C/5% CO_2_. Cells were washed twice with medium and placed back at 37°C/5% CO_2_. 24 hours later, cells were fixed with 2% formaldehyde/0.1% glutaraldehyde in PBS for 15 minutes at 37°C followed by a 15 minute blocking with PBS/1% (v/v) FCS at 25°C. Cells were permeablized with PBS/1% (v/v) FCS/0.1% (w/v) saponin for 15 minutes at 25°C. LCMV infection was detected using mAb 113 anti-LCMVNP [30] (1:200) in PBS/1% (v/v) FCS/0.1% (w/v) saponin for 1 hour at 25°C. Following 2 washes, goat anti-mouse IgG FITC conjugated secondary antibody was applied for 45 minutes at 25°C. Fluorescence microscopy using a 5X objective was used to count NP+ cells. LCMV β-gal pseudotypes were detected using a β-gal staining kit (Invitrogen, K1465-01).

### *In vivo *vector administration

Adult BALB/c mice received the following doses of vector via tail vein: FIV-L260F, 4 × 10^7 ^TU; FIV-WE54, 8 × 10^7 ^TU; FIV-GP64, 4 × 10^7 ^TU. Neonatal BALB/c mice (day 2 of life) were injected with 100 μl of centrifuge concentrated FIV vector via the facial vein using a 30-gauge needle and a 1 ml syringe over 20 seconds. Vector was not delivered hydrodynamically. The delivered dose of vector varied depending on the titer of the concentrated virus. For bioluminescence studies, FIV-WE54-Luc vector titers allowed for the highest delivered doses of ~5.1 × 10^7 ^TU, followed by FIV-L260F (1.2 × 10^7 ^TU), and FIV-GP64 (1.0 × 10^7 ^TU). For tissue staining studies, the approximate transducing units delivered were 5.0 × 10^6 ^for FIV-WE54-ntLacZ, 4.0 × 10^6 ^for FIV-L260F-ntLacZ, and 2.0 × 10^7 ^for FIV-GP64-ntLacZ. The vectors administered *in vivo *are presented in Table 1. The University of Iowa Institutional Animal Care and Use Committee approved this study.

**Table 1:** Vector Doses Administered In Vivo*

**Adult mice (luciferase vectors)**^**++**^	
FIV-GP64, 4 × 10^7 ^TU	

FIV-WE54, 8 × 10^7 ^TU	

FIV-L260F, 4 × 10^7 ^TU	

**Neonatal mice**^**++**^	

Luciferase Vectors	β-Galactosidase Vectors

FIV-GP64, 1.0 × 10^7 ^TU	FIV-GP64, 2.0 × 10^7 ^TU

FIV-WE54, 5.1 × 10^7 ^TU	FIV-WE54, 5.0 × 10^6 ^TU

FIV-L260F, 1.2 × 10^7 ^TU	FIV-L260F, 4.0 × 10^6 ^TU

*Presented as total number of transducing units (TU) delivered to each animal.

^**++**^Vectors administered to adult mice via tail vein. Vectors delivered to neonatal mice on day 2 of life via the facial vein (100 μl of centrifuge concentrated vector). The most concentrated preparations were used for each vector.

### Bioluminescence imaging

Following FIV-Luc delivery, *in vivo *luciferase expression was visualized using bioluminescence imaging as described [24]. At the time points indicated, D-luciferin (100 μl/10 g of body weight (15 mg/ml in PBS) (Xenogen)) was delivered intraperitoneally to animals using a 26-gauge needle, then mice were placed under 2-3% isoflurane anesthesia. Five minutes after D-luciferin injection, animals were placed in the Xenogen IVIS-200 imaging cabinet (Alameda, CA) and imaged using a Xenogen IVIS charge-coupled device (CCD) camera while anesthetized. Imaging data were analyzed and signal intensity quantified using Xenogen Living Image software.

### Luciferase assays

Three weeks post transduction, animals were euthanized and the heart, lung, liver, spleen, and kidneys harvested following vascular perfusion with PBS. Tissues were homogenized in Tropix lysis buffer (Applied Biosystems) then centrifuged for 10 minutes at 18,000 × g. Luciferase assays were performed following manufacturer's instructions (Promega, E1501) and quantified using a PharMingen Monolight 3010 luminometer. Samples were normalized for protein content by Lowry assay.

### Tissue sectioning and X-gal staining

Tissues harvested from animals 3 weeks post-injection were embedded in Tissue-Tek O.C.T. compound and frozen at -80°C. 8 μm sections were made using a Microm Cryostat I (HM 505E). Slides were fixed for 10 minutes at 25°C in 0.5% (v/v) glutaraldehyde/PBS then washed twice in 1 mM MgCl_2_/PBS for 10 minutes. Slides were X-gal stained in Coplin jars for 10 minutes at 37°C then washed immediately in 1 mM MgCl_2_/PBS. Postfixation occurred in 0.5% (v/v) glutaraldehyde, 10% (v/v) formalin/PBS for 10 minutes at 25°C. Slides were counterstained 1 minute with nuclear fast red then coverslipped. Quantification of β-gal expression was performed utilizing ImagePro Plus 5.1 (Media Cybernetics) examining 20X and 40X magnification images as previously reported [24]. Three images per slide were quantified and results averaged from at least 3 slides per tissue. Manual counting of 40X magnification images confirmed the accuracy of automated measurements of transduction efficiency. Cell types were determined by examining nuclear and cellular morphology.

## Results

### Developmental expression of α- and β-DG in liver tissue

**Figure 1:**
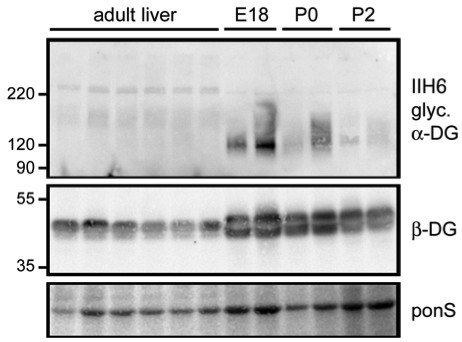
**Developmental expression of dystroglycan proteins in mouse liver**. WGA enriched glycoproteins from livers taken from pooled E18 embryos, postnatal day zero mice (P0), postnatal day 2 mice (P2), and adult mothers were separated on SDS PAGE. Immunoblots were performed with IIH6 antibody recognizing the glycosylated form of α-DG or a polyclonal antibody for β-DG. Ponceau S stained portions of the blot are shown as a protein loading control.

The availability of target receptors influences the efficiency of gene transfer in neonatal and adult tissues. We hypothesized that changes in the levels of α-DG or other viral receptors expressed in newborn versus adult liver would influence the gene transfer efficiency with LCMV pseudotyped lentiviral vectors. To test this hypothesis, we isolated WGA enriched glycoproteins from neonatal livers and compared the expression of α- and β-DG by western blotting (Figure 1). The IIH6 antibody for α-DG recognizes the glycosylated form of α-DG that binds with high affinity to LCMV and laminin. Liver from E18 embryos and early postnatal liver showed high levels of IIH6 reactive α-DG, while in adult liver, IIH6 reactive α-DG was barely detected. In addition, β-DG levels were also high in late embryonic/neonatal livers compared to adult animals. Interestingly, although the β-DG levels remained high in P0 and P2 embryos, the level of IIH6 reactive α-DG appeared to progressively decrease over this time window. This could either reflect a loss in the α-DG protein or a decrease in glycosylation efficiency with an overall effect of loss of functional α-DG on the cell surface.

### LCMV glycoproteins efficiently pseudotype feline immunodeficiency virus

We and others have successfully pseudotyped feline immunodeficiency virus (FIV)-based lentiviral vectors with envelope glycoproteins from the baculovirus [24], rhabdovirus [23,31], coronavirus [32], alphavirus [31], and filovirus [33] families. Oncoretroviral and HIV-based lentiviral gene transfer vectors pseudotyped with the LCMV envelope yield titers similar to VSV-G while displaying broad tissue tropism [1]. Utilizing a recombinant LCMV envelope (LCMV WE-HPI) that allows for efficient processing and cell surface expression [21], we achieved concentrated FIV titers of ≥10^8 ^TU/ml.

### Entry of LCMV pseudotyped FIV depends on fusion in a low pH endosomal compartment

**Figure 2:**
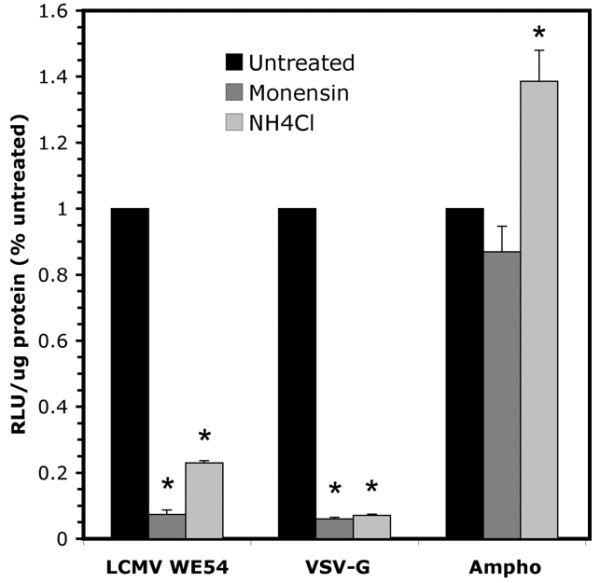
**LCMV pseudotyped FIV requires low pH endosomes for efficient transduction**. A549 cells were pretreated with 8 μm monensin or 10 mM NH_4_Cl to prevent endosomal acidification followed by application of FIV vectors pseudotyped with LCMV WE54, VSV-G, or amphotropic envelope. Transduction efficiencies were measured by β-gal assay and normalized to cells that received no pretreatment. * indicates *P *value ≤ 0.05 compared against untreated control (n = 9). Standard errors are denoted.

Borrow el al. [27] previously demonstrated the pH dependence of the LCMV entry process. Used as a control in their experiments, VSV is known to fuse with cells in a low pH endosome. We sought to demonstrate that FIV pseudotyped viruses retain their wild-type entry characteristics. As shown in Figure 2A, pretreatment of A549 cells with either the ionophore monensin, which prevents endosomal acidification, or the weak base ammonium chloride inhibited transduction with FIV vectors pseudotyped with LCMV WE54 and VSV-G. Monensin pretreatment (8 μM) resulted in a 93% decrease in transduction by both LCMV and VSV-G pseudotyped vectors when compared to transduced cells that received no pretreatment. Similarly, ammonium chloride pretreatment caused a 77% decrease in transduction of FIV-LCMV-WE54 and a 93% transduction decrease of FIV-VSV-G. Negative control amphotropic MLV envelope pseudotyped FIV displayed no transduction inhibition by monensin or ammonium chloride. Fusion of the amphotropic envelope occurs at the cell surface and displays pH independence [34]. From these findings, we conclude that the LCMV-WE54 envelope glycoproteins maintain their native entry and fusion properties when displayed on the FIV lentivirus, effectively changing the route of entry that FIV normally takes [35]. These data agree with previous findings by Sinn *et al. *demonstrating that some envelope glycoproteins retain native entry mechanisms following lentiviral pseudotyping [24].

### A single point mutation to LCMV GP1 alters its α-DG affinity

**Figure 3:**
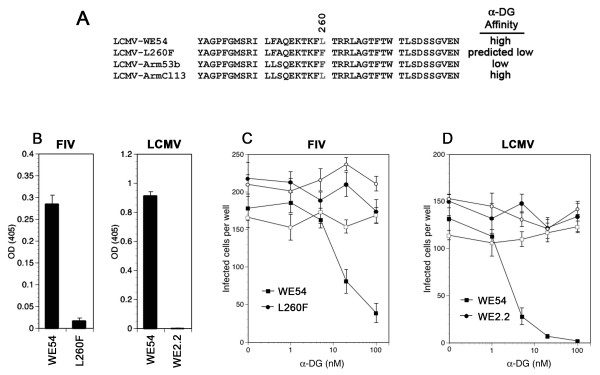
**The LCMV L260F mutation significantly reduces its affinity for α-DG**. (A) GP1 of LCMV WE54 was mutated at amino acid position 260 to produce a low affinity α-DG binding envelope (LCMV L260F) similar to the wild-type envelope of LCMV Arm53b. Leucine at position 260 results in high affinity binding to α-DG; phenylalanine reduces its affinity. (B) LCMV pseudotypes and wild type virus were compared for their affinity to immobilized α-DG in an ELISA-based assay detecting bound virions. (C) Increasing amounts of soluble α-DG were preincubated with FIV-WE54 (filled squares) or FIV-L260F (filled circles) to neutralize transduction measured by counting positive cells per well. BSA preincubation was used as a control (open symbols). Wild-type LCMVs with known high (WE54) or low (WE2.2) affinity for α-DG were used as controls for contrast with pseudovirions and depicted in (B, right panel) and (D). n = 3. Standard deviations are plotted.

To investigate the tropism of LCMV WE54 pseudotyped vectors with the knowledge that high and low α-DG affinity arenaviruses display unique tropisms, we set out to generate a pseudotyped vector possessing low affinity for α-DG. To alter the α-DG affinity of LCMV WE54 we mutated residues previously identified as responsible for α-DG binding (Figure 3A). The serine residue at position 153 of GP1 was changed to phenylalanine, which mimics the WE2.2 strain mutation, or the leucine at position 260 was mutated to a phenylalanine, imitating the Arm53b to Cl13 mutation. LCMV pseudotyped with WE54 S153F yielded low titers (≤10^3 ^TU/ml), whereas the L260F mutation (Figure 3A) resulted in a modest loss of titer, approximately a half log (~8 × 10^7 ^TU/ml, n = 6) compared to parental FIV-WE54 (1 × 10^8 ^- 4 × 10^8 ^TU/ml, n = 6). Pseudotyping FIV with the LCMV WE54 double mutant containing the S153F and L260F mutations also resulted in substantial loss of vector titer and was unsuitable for study.

To verify altered affinity for α-DG, we performed a series of competition assays. The virus binding affinity was determined by immobilizing α-DG in 96-well microtiter plates followed by incubation with either WE54 or L260F pseudotyped FIV. Wild-type LCMV WE54 and WE2.2 viruses were used as controls. At equivalent viral loads, FIV-WE54 bound to α-DG ~13 times more effectively than FIV-L260F suggesting that the point mutation altered its α-DG affinity (Figure 3B). As expected, wild-type WE54 demonstrated strong affinity while WE2.2 displayed little to no affinity (Figure 3B). FIV-WE54 binding, measured by optical density (OD), was approximately one-third the OD of its wild-type counterpart.

To further demonstrate that the LCMV WE54 and L260F envelopes differ in their α-DG affinity, we used neutralization assays to ask whether soluble α-DG inhibited transduction by preventing receptor binding. With increasing concentrations of soluble α-DG, vector neutralization was seen with LCMV WE54 pseudotyped FIV (Figure 3C) and wild-type LCMV WE54 (Figure 3D). No neutralization was observed from FIV-L260F or wild-type LCMV WE2.2 as expected. Incubating virus with increasing concentrations of BSA did not change transduction of any vector. The concentration of soluble α-DG required to produce a 50% reduction in binding (C_50_) for FIV-WE54 was ~20 nM whereas wild-type LCMV WE54 C_50 _was ~2-3 nM. From these findings, we conclude that the GP1 L260F mutation of the LCMV WE54 strain envelope significantly decreases α-DG affinity in the context of a FIV lentiviral pseudotype.

### *In vivo *delivery of LCMV pseudotyped lentiviral vectors

**Figure 4:**
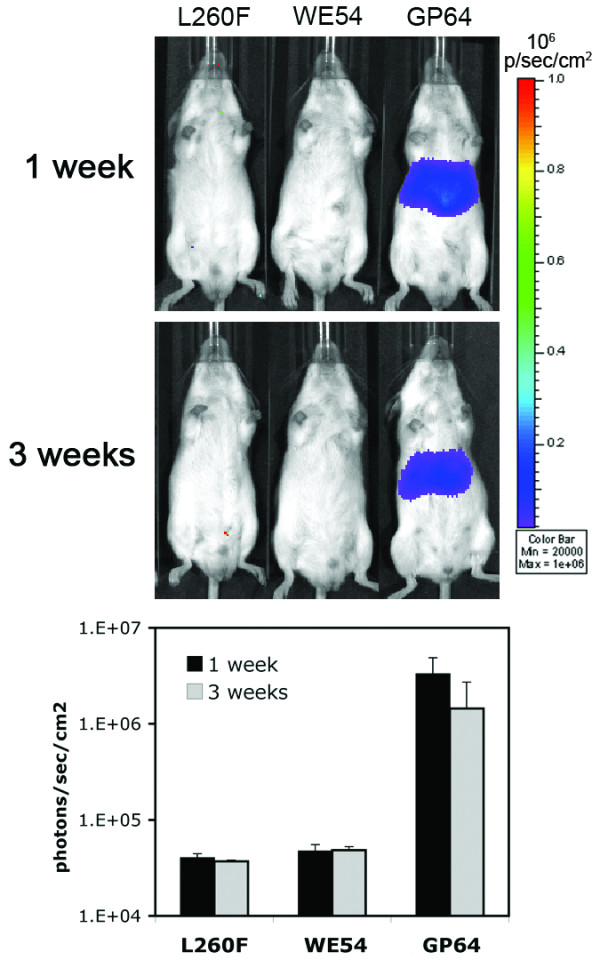
**LCMV pseudotyped FIV fails to transduce adult mice tissues following tail vein injection**. 6-8 week old mice were injected with FIV pseudotypes (LCMV L260F, LCMV WE54, or GP64 left to right) and subsequently imaged to detect bioluminescence 1 and 3 weeks post-injection. Bioluminescence intensities were measured and means plotted with standard error. n = 6 animals/group.

To determine the *in vivo *tropism of LCMV pseudotyped FIV, vector was delivered to 6-8 week old adult mice either locally to the respiratory epithelia [24] or systemically via tail vein [31]. Attempts to transduce the respiratory tract in adult mice with FIV-WE54 and FIV-L260F yielded undetectable luciferase signals in enzyme assays and bioluminescence imaging following intranasal or intratracheal delivery (data not shown). Intravenous vector delivery to adult mice also yielded undetectable levels of luciferase expression (Figure 4). In contrast, FIV pseudotyped with the baculovirus GP64 envelope successfully transduced adult murine tissues following each delivery route (Figure 4 and data not shown), as shown previously [26]. The lack of transduction in adult murine tissues may be due to the developmental regulation of α-DG (the receptor for the WE54 strain of LCMV), a consequence of post-translational modifications of α-DG, or related to other post receptor binding steps [36]. Since α-DG expression is reported to be most abundant in fetal and neonatal tissues [7-9] and Figure 1, we next tested LCMV tropism in neonatal mice.

**Figure 5:**
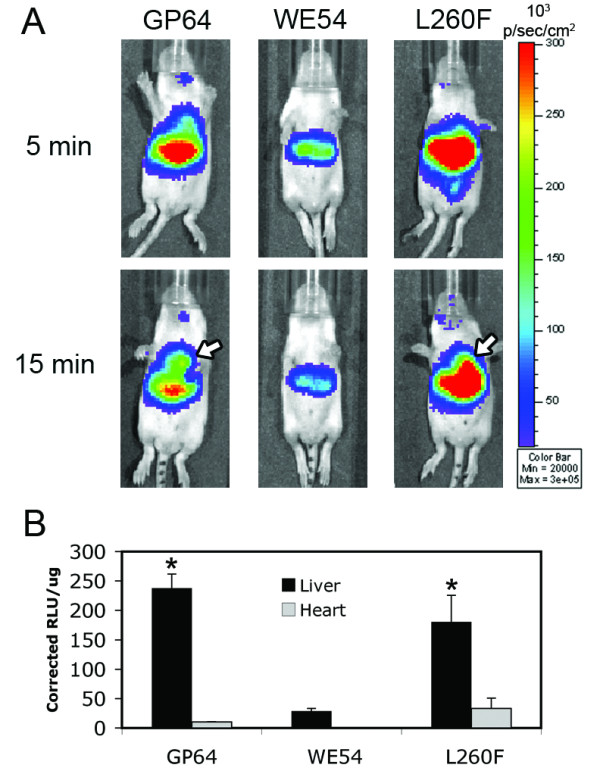
**Neonatal delivery of FIV-L260F transduces the liver and heart**. One week after facial vein injection of pseudovirions, mice were imaged utilizing a CCD camera. (A) Representative photos of mice 1 week postinjection are depicted following injection with FIV-GP64, FIV-WE54, and FIV-L260F. Waiting an additional 10 minutes after IP delivery of D-luciferin allowed for luciferase expression to be detected from the hearts (arrows) in animals transduced with FIV-GP64 and FIV-L260F, while the hearts of FIV-WE54 transduced animals displayed no detectable signal. (B) Three weeks postinjection, the heart, lungs, liver, spleen, and kidneys were harvested and luciferase assays conducted. Expression was detected exclusively from liver tissue (black bars) for all pseudoviruses tested and from hearts (gray bars) for the GP64 and L260F pseudoviruses. Standard errors are plotted and * denotes statistical significance (*P *≤ 0.05) compared to FIV-WE54 livers. n = 5 for FIV-GP64 and FIV-WE54, 8 for FIV-L260F.

We delivered FIV lentiviruses pseudotyped with the LCMV WE54, LCMV L260F, or GP64 envelopes systemically via the facial vein to neonatal BALB/c mice. Vectors expressed a firefly luciferase reporter directed by the RSV promoter and expression was assessed 1 and 3 weeks post-injection. FIV pseudotyped with the WE54 envelope (high affinity for α-DG) transduced tissues less efficiently than FIV-GP64 at 1 week (Figure 5A). Photon emission appeared to be predominantly localized to the liver with occasional weak signal evident near the injection site. In contrast to FIV-WE54, animals transduced with the FIV-L260F vector displayed significantly better expression (Figure 5A). Interestingly, waiting an additional 10 minutes after luciferin administration enhanced signal over the heart for both FIV-GP64 and FIV-L260F (arrows, Figure 5A, arrows). Similar observations were made 3 weeks post injection (data not shown). At the 3 week time point, following CCD imaging, the heart, lung, liver, spleen, and kidneys were harvested. Luciferase assays were performed to assess tissue tropism and expression. Transgene expression was highest in the livers of all animals and no expression was detected in the spleen, lungs, or kidneys. Luciferase assays revealed that FIV-L260F yielded ~10-fold higher expression (RLU/μg) in the liver compared to FIV-WE54 (Figure 5B). FIV-L260F displayed ~5-6 times higher expression in the liver compared to the heart. LCMV WE54 displayed no heart transduction. The positive control GP64 pseudotyped FIV displayed expression from both the liver and heart.

**Figure 6:**
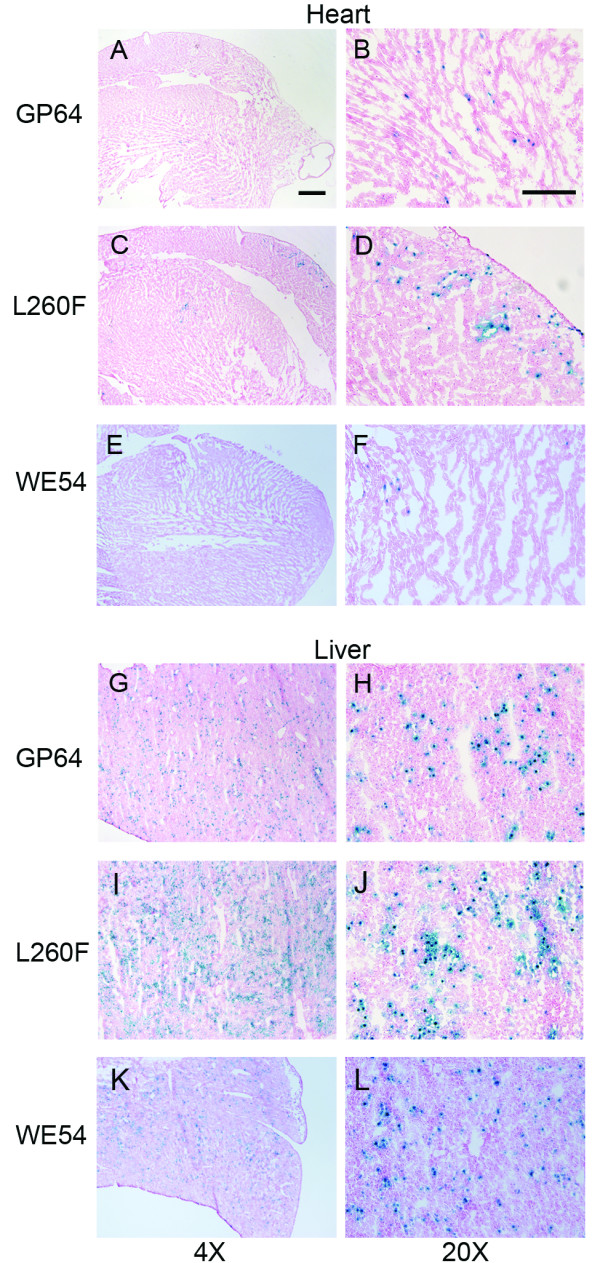
**Localization of neonatal transduction in murine tissues**. FIV vectors expressing the LacZ gene were injected into neonatal mice. Three weeks post-injection, organs were removed and cryosectioned (8 μm). 4X and 20X magnification images are shown for FIV-GP64 transduced hearts (A-B) and livers (G-H) following X-gal staining. Similar images are displayed for FIV-L260F transduced hearts (C-D) and livers (I-J) and FIV-WE54 transduced hearts (E-F) and livers (K-L). Spleen and kidneys of vector-transduced animals were negative. Slides were counterstained with nuclear fast red. Scale bar in A is 500 μm; B is 200 μm. n = 3.

Neonatal mice next received FIV vectors expressing nuclear targeted β-gal to examine the tissue and cellular distribution, as well as to estimate the transduction efficiency. Three weeks post-injection, animals were sacrificed and tissues fixed, cryosectioned, and X-gal stained. Heart tissue from FIV-GP64 (Figures 6A, B) and FIV-L260F (Figures 6C, D) treated animals displayed β-gal positive cells, while little signal was observed with FIV-WE54 (Figures 6E, F). Additionally, transduced livers from all animals displayed uniform staining throughout the tissue (Figures 6G-L). Variability in transduction efficiency was observed between individual animals injected with the same vector, likely representing differences in the effective delivered doses. The FIV-L260F transduction efficiency ranged from 5-23% positive cells in liver sections and 1-7% positive cells in the heart. The cardiac transduction was patchy with predominate expression observed in cardiomyocytes. By morphological criteria, hepatocytes were the predominant target of liver cell transduction by all envelope glycoproteins tested (Table 2).

**Table 2:** Liver transduction with FIV pseudotypes

		Transduced cell type, %	
		
Vector	% Transduction^*^	Hepatocytes	Non-hepatocytes
FIV-GP64-ntLacZ	5.1 (0.6)	99.9	0.1
FIV-L260F-ntLacZ	14.5 (1.2)	99.9	0.1
FIV-WE54-ntLacZ	6.7 (0.8)	99.9	0.1

* Mean (+/- SE)

**Figure 7:**
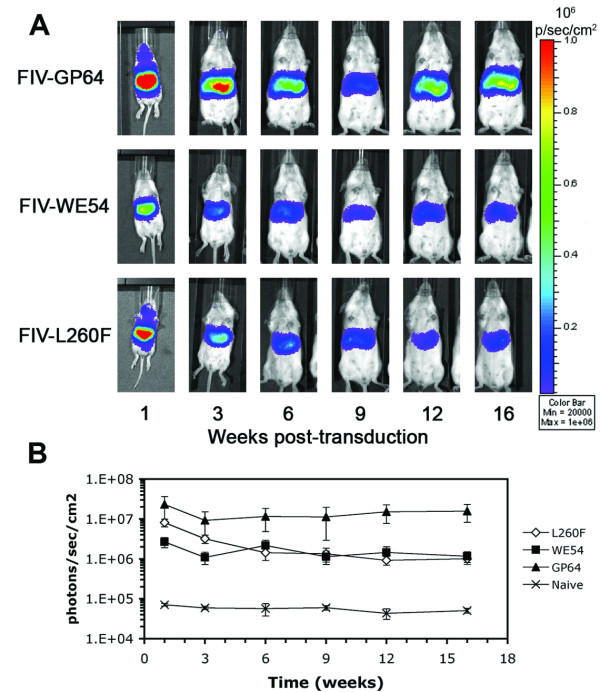
**LCMV pseudotyped FIV transduction persists *in vivo***. Luciferase expression was monitored up to 16 weeks following neonatal injection and signal intensities were quantified using Living Image 2.50 software. 100 μl of FIV-GP64 (an average of 1.0 × 10^7 ^TU), FIV-WE54 (~5.1 × 10^7 ^TU), or FIV-L260F (~1.2 × 10^7 ^TU) were injected via the facial vein 40-60 hours postnatally. Naïve mice received no injection. Images shown are representative of each condition. Animal groups n = 11 for FIV-GP64, 13 for FIV-WE54, and 17 for FIV-L260F. Standard errors are depicted with the average signal intensities.

Subsequent cohorts of neonatal animals received the same three vectors intravenously so that luciferase expression could be monitored for persistence. Animals were imaged 1, 3, 6, 9, 12, and 16 weeks post-injection (Figure 7A). After an initial decline at 1 to 3 weeks, expression stabilized and remained relatively constant throughout the duration of the 16 week experiment. As shown in Figure 7B, starting with an average luciferase signal intensity of 8.1 × 10^6 ^photons/sec/cm^2 ^(+/- 1.8 × 10^6 ^photons/sec/cm^2 ^SE) at the 1 week post-injection time-point, FIV-L260F expression after 16 weeks averaged 1.0 × 10^6 ^photons/sec/cm^2 ^(+/- 2.8 × 10^5 ^photons/sec/cm^2 ^SE). FIV-WE54 expression also persisted and at the 16 week time-point averaged above 10^6 ^photons/sec/cm^2^. Expression from control FIV-GP64 was stable at ~10^7 ^photons/sec/cm^2^. Over the 16 week time course, expression was almost always detected from the liver area. At early time points it was not uncommon to detect signal near the site of injection; this signal disappeared at later time points.

## Discussion

We successfully developed a recombinant LCMV envelope glycoprotein with altered affinity for its known receptor, α-DG and used this to pseudotype FIV. Other strains of LCMV and additional members of the arenavirus family are α-DG receptor independent and typically display unique tropisms. Our modified WE54 envelope was generated using the knowledge that in the LCMV Arm53b strain, a leucine to phenylalanine substitution at position 260 of GP1 alters α-DG affinity. Incorporating this mutation into the LCMV WE54 glycoprotein did not negatively affect lentivirus titer and resulted in altered receptor affinity. The LCMV L260F envelope efficiently transduced neonatal murine hepatic and cardiac tissues despite somewhat titer-limited lower delivered doses, and conferred stable expression.

Differences arose when comparing wild-type LCMV to lentiviral pseudotypes with respect to their α-DG binding affinities and neutralization properties. Wild-type LCMV WE54 bound approximately three times greater to immobilized α-DG than its pseudotyped counterpart (Figure 3B). There are multiple possible explanations for this observation. LCMV envelope glycoproteins normally form homotetramers on the virion surface. However, the HIV-1 and SIV lentiviruses have been demonstrated by electron tomography to possess as few as 8-10 trimers per virion [37]. Thus, glycoproteins displayed on the surface of FIV particles may not mirror presentation on native LCMV virions. This could reduce α-DG binding and might also decrease recognition by the monoclonal antibody used against GP2. A difference in the number of glycoproteins displayed on a virion may also lead to a disparity between wild-type and pseudotype receptor binding affinity. Regardless, the results of the viral neutralization and α-DG binding assays confirm that FIV-WE54 binds significantly better than vector with the LCMV L260F envelope.

Both LCMV envelopes transduced the neonatal liver following systemic delivery. Expression from the lentiviral vectors persisted at least 4 months (duration of the experiment). These same pseudotypes poorly transduced adult liver following tail vein injection while FIV-GP64 transduced the livers of both adult and neonatal mice. FIV-GP64 transduction, measured by bioluminescence, was slightly higher than LCMV pseudotypes following neonatal delivery. In contrast, FIV-WE54 and FIV-L260F failed to transduce adult mouse tissues following intravenous, intranasal, or intratracheal delivery. Of note, the LCMV WE54 pseudotype only transduced neural stem cells/progenitors following striatal injections in adult mouse brain, while the L260F pseudotype failed to transduce cells following striatal injections ([38] and personal communication from Dr. Beverly Davidson). The difference in transduction efficiency between neonatal and adult animals suggests that arenavirus receptor expression varies considerably during development. Indeed our studies of α-DG expression neonatal vs adult liver tissues revealed a much higher level of expression of the IIH6 reactive form of alpha-dystroglycan perinatally compared to adult animals. We also did not detect any significant gene transfer by FIV-WE54 in skeletal muscle even though α-DG is abundantly expressed in these tissues.

The glycan(s) present on α-DG that reacts with IIH6 is thought to be critical for LCMV binding and laminin binding. However, most of the α-DG in the neonatal liver was of a lower molecular weight (~120 kDa) than the α-DG glycoform that typically found in adult skeletal muscle (156 kDa), but comparable to what is found in brain dystroglycan (~120 kDa) [20]. The α-DG receptor, which the WE54 envelope binds with high affinity, exhibits higher expression in late embryonic and neonatal stages and declines thereafter [8,9]. Interestingly, the infection patterns of developing brain by LCMV are also strikingly dependent on the age of the animal [39]. Therefore, although FIV-L260F shows a lower affinity for α-DG, the unique glycosylation patterns in neonatal liver may provide a virus-glycan interaction that favors low affinity arenavirus binding [18,36]. There may also be developmentally regulated cell type specific differences in entry steps that occur after receptor binding, the tropism of the vector may be strongly affected by competition of the virus with other ligands that bind α-DG including laminin and other basement membrane proteins that are developmentally regulated. More studies are also needed to identify the potential alternative receptor for the α-DG low affinity arenaviruses, specifically of the Old World arenaviruses. This would allow characterization of the expression patterns of this receptor to determine if its developmental regulation contributes to arenavirus tropism.

Another possible explanation for the differing transduction efficiencies in neonatal and adult mice may relate to hepatocellular proliferation rates. Park *et al. *demonstrated improved lentiviral transduction of hepatocytes *in vivo *following cell cycle progression [40]. Further support of this hypothesis came from correlating vector transduction efficiencies and BrDU labeling in mice of different ages [41]. It is known that the rates of hepatocyte proliferation are higher in newborn than adult animals [42,43]. A requirement for cell proliferation may also help explain the relatively low efficiency of LCMV pseudotyped vector tropism for adult striated muscle cells, which have high α-DG expression, but lack nearly any detectable mitotic activity under normal conditions. However, other physiological differences between neonatal and adult mice cannot be ruled out.

The FIV-L260F envelope conferred higher transgene expression than FIV-WE54 in neonatal mice initially (1 and 3 weeks post-injection) and subsequently displayed similar levels of expression for the remaining 13 weeks of observation (Figure 7). This is despite an average delivered vector dose approximately 20% of FIV-WE54. Additionally, FIV-L260F displayed a unique tropism that included cardiomyocyte transduction. A general observation was that heart transduction from FIV-L260F was primarily located in ventricular free walls, although rare transduction was also observed in the interventricular septum. Most expression was found in the outer 300 μm of the myocardium. In contrast, FIV-GP64 displayed more uniform transduction across the full thickness of the myocardium. These observations suggest coronary circulation as the route of delivery to these areas rather than transduction through the endocardium.

## Conclusions

LCMV pseudotyped FIV may have applications for delivery of genes to hepatocytes [1] and cardiomyocytes during development for experimental or therapeutic applications. Neonatal gene transfer has several posited advantages and has received increased interest with achievements utilizing oncoretroviral and lentiviral vectors [44-51]. Hereditary disorders that cause specific organ damage prenatally or neonatally could be treated early before irreversible damage. The neonatal immune system is less mature, and may allow enhanced transduction and facilitate transgene tolerance [52,53]. A practical advantage of neonatal gene transfer is that an increased delivered vector/body mass can be achieved experimentally, perhaps allowing for transduction of a larger number of the cells in a tissue, and increasing the likelihood of progenitor cell targeting when integrating vectors are used. In summary, LCMV L260F pseudotyped FIV has a reduced affinity for α-DG receptor and may have applications as a neonatal gene transfer vector for cardiac and hepatic tissues via intravenous delivery.

## List of Abbreviations

Discussed in text.

## Competing interests

The authors declare that they have no competing interests.

## Authors' contributions

DD participated in the design of the study, carried out the experiments, and drafted the manuscript. DD, LX, DM, and SK performed experiments and participated in discussions. PM designed this study and edited the manuscript. All authors read and approved the final manuscript.
